# Anesthesia for pulmonary veno-occlusive disease: the dilemma and what we should know as anesthesiologists: A case report

**DOI:** 10.1097/MD.0000000000021517

**Published:** 2020-07-24

**Authors:** Theng Wai Foong, Pei Shan Tan, Swapna Thampi, Ashokka Balakrishnan

**Affiliations:** a Department of Anesthesia and Surgical Intensive Care, National University Hospital; b Yong Loo Lin School of Medicine; c Centre For Medical Education, National University of Singapore, Singapore.

**Keywords:** anagrelide, combined spinal-epidural, pulmonary hypertension, pulmonary veno-occlusive disease, vasodilator

## Abstract

**Introduction:**

Pulmonary veno-occlusive disease (PVOD) is a rare form of pulmonary hypertension. It is often underdiagnosed or misdiagnosed as idiopathic pulmonary arterial hypertension (PAH). Inappropriate treatment may cause worsening of symptoms which may lead to fatal outcomes. Anesthetic considerations and management for pulmonary hypertension are well described, but few anesthesiologists are aware of the entity of PVOD and its management.

**Patient concerns:**

We report a case of PVOD in a 73-year-old female who was on concurrent aspirin and anagrelide, requiring emergent open femoral hernia repair.

**Diagnosis:**

PVOD and incarcerated femoral hernia

**Intervention:**

Combined spinal-epidural (CSE) was performed to enable the surgery.

**Outcome:**

Surgery was completed successfully under central neuraxial anesthesia and the patient remained stable and comfortable throughout, avoiding the need for general anesthesia. Due to the concurrent aspirin and anagrelide therapy, significant bleeding from the CSE puncture site was observed immediately post-operatively. This was resolved with external manual compression and withholding the aspirin and anagrelide. Patient remained well without neurological deficit and was discharged postoperative day seven.

**Lessons:**

It is important to differentiate PVOD from PAH due to the controversial use of pulmonary vasodilators in PVOD. Pulmonary vasodilator is commonly used to treat acute pulmonary hypertension in PAH but its usage may lead to pulmonary edema in patients with PVOD. Hence, with no ideal treatment available, the avoidance of general anesthesia is crucial to prevent acute pulmonary hypertensive crisis in patient with PVOD. However, this needs to be weighed against the elevated risk of central neuraxial bleeding when performing a CSE in a patient on concurrent aspirin and anagrelide therapy. Calculated decision-making considering the risks and benefits of all alternatives should be carried out in such a scenario, and measures should be taken in anticipation of the potential consequences of the eventual decision.

**Conclusion:**

It is important to differentiate PVOD from PAH. PVOD has unique anesthetic considerations due to the controversial use of pulmonary vasodilators. This case also emphasizes the importance of active anticipation of potential issues and adequate follow up.

## 1 Introduction

Pulmonary veno-occlusive disease (PVOD) is a rare form of pulmonary hypertension with preferential remodeling of pulmonary venules. It is classified as a subgroup of group 1 pulmonary arterial hypertension (PAH) under the unified entity of PVOD/pulmonary capillary hemangiomatosis (PCH).^[1]^ The prevalence of PVOD is estimated to be 1 to 2 cases per million population per year. Classical presentation includes exertional dyspnea, lethargy with signs and symptoms of right heart failure. Severe reduction in diffusing capacity of the lung for carbon monoxide (DLCO) to <50% of predicted value is common in PVOD and poses great challenges in anesthesia management. It is important to differentiate PAH from PVOD as vasodilator drugs that are used to treat idiopathic PAH may lead to life-threatening pulmonary edema in patients with PVOD.^[2,3]^

## 2 Case report

A 73-year-old, 41 kg and 148 cm, ethnic Chinese female with newly diagnosed PVOD presented with an incarcerated femoral hernia requiring surgical intervention. She was also on concurrent aspirin and anagrelide, a phosphodiesterase III inhibitor, for treatment of Polycythemia Vera.

She was admitted for symptoms of decompensated heart failure with lower limb swelling, intermittent palpitations, and progressive exertional dyspnea. Her chest x-ray on admission showed pulmonary edema and cardiomegaly and an arterial blood gas revealed hypoxia with a PaO2 of 63 mm Hg on room air. An urgent transthoracic echocardiography was performed revealing a significantly elevated pulmonary artery systolic pressure of 74 mm Hg associated with severe functional tricuspid valve regurgitation and abnormal ventricular septal motion. Pulmonary function test showed a reduced DLCO of 34% but otherwise normal spirometry. A series of investigations including computed tomography-pulmonary angiography to rule out pulmonary embolism, right heart cardiac catheterization, and high-resolution computed tomography of the thorax to rule out intrinsic lung disease were subsequently undertaken leading to the diagnosis of PVOD.

During the same admission, she presented with a painful incarcerated femoral hernia. A multi-disciplinary discussion was held between the cardiologist, surgeon, and anesthesiologist regarding medical and surgical options, perioperative risk stratification and risk counseling. A consult to the cardiothoracic team was made regarding the option for extracorporeal membrane oxygenation support in the event of cardiopulmonary collapse during anesthesia, however, the patient was deemed unsuitable in view of her age and irreversibility of PVOD.

A consensus was reached to proceed with an open left femoral hernia repair with the possibility of conversion to laparotomy and bowel resection under regional anesthesia with a lumbar combined spinal-epidural (CSE). She had received her last dose of anagrelide 8 hours before surgery and aspirin was withheld 24 hours before the surgery. Her platelet count was 529 × 10^9^ L^−1^ and her coagulation profile was normal.

During the procedure, standard American Society of Anesthesia monitors were placed and intra-arterial cannula was inserted for continuous blood pressure monitoring. Transcutaneous defibrillator pads were placed on the patient due to the increased risk of cardiovascular collapse. Supplemental oxygen was provided via a Hudson facemask at 5 L min^−1^. End tidal carbon dioxide and respiratory rate monitoring were established via a carbon dioxide sampling line through the face mask. The patient's baseline vitals included a SpO2 of 91% on room air, which increased to 100% with supplemental oxygen, a blood pressure of 160/78 mm Hg and a heart rate of 72 beats per minute.

Lumbar CSE was performed under aseptic technique. It was a technically challenging procedure due to the presence of lumbar scoliosis with successful insertion at the third attempt. The intrathecal dose of 0.9 mL of isobaric bupivacaine 0.5% (4.5 milligrams) and 15 micrograms of fentanyl was administered, and 4 mL bolus lignocaine 1.5% with sodium bicarbonate was administered via the epidural catheter. No sedation was required and the patient tolerated the procedure well. A complete motor block of the lower limbs and a sensory level of T10 were successfully established before the start of surgery. An additional 3 mL lignocaine 1.5% was administered through the epidural catheter after 45 minutes and surgery was completed in 70 minutes. Patient remained hemodynamically stable throughout. Additional analgesia was provided with infiltration of 10 mL bupivacaine 0.5% at the surgical site.

**Figure 1:**
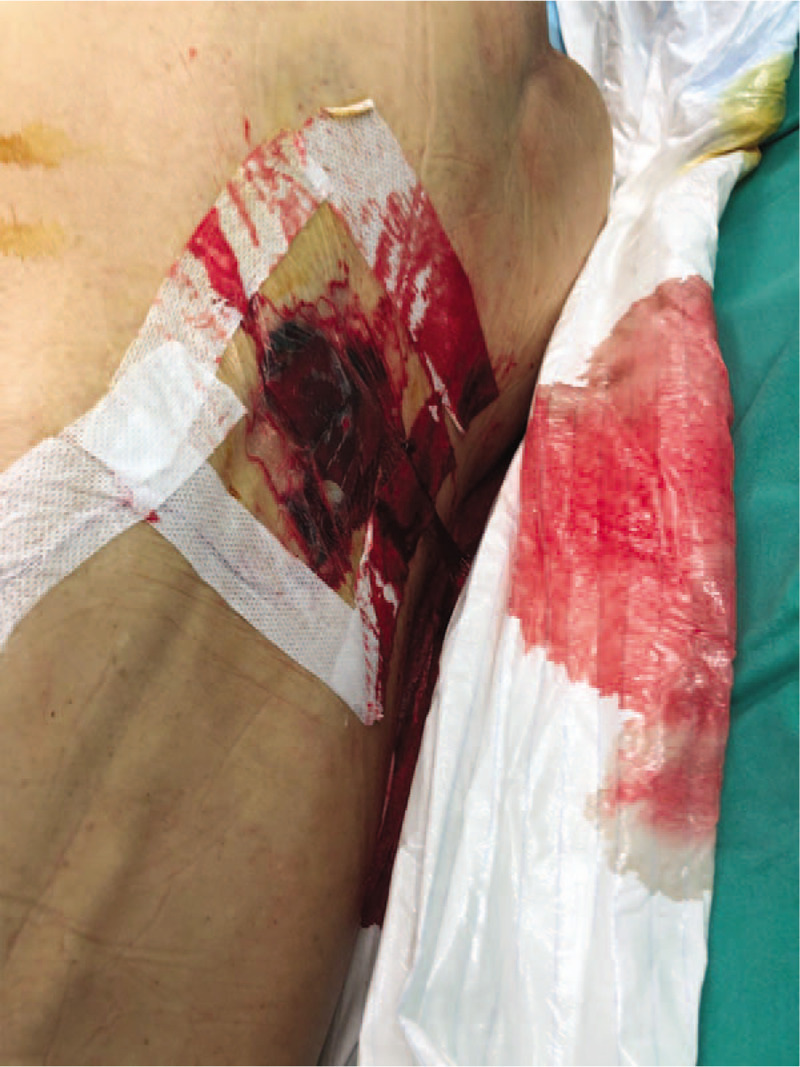
Bleeding at combined spinal-epidural puncture site after the surgery.

The patient's epidural catheter was removed immediately after the surgery, however, significant bleeding at the puncture site was noted before removal (Fig. 1). Sustained pressure was applied over the catheter insertion site after catheter removal. The patient was then observed in the post anesthesia care unit for 1 hour until full neurological recovery was established. No further bleeding from the catheter insertion site was noted during this period. She was discharged well when stable to the surgical high dependency unit.

Anagrelide was held off for 72 hours following the CSE and acute pain team reviewed the patient twice a day subsequently. Relevant care teams were informed on potential red flag symptoms and signs that could indicate development of central neuraxial hematoma. Her coagulation profile remained normal postoperative. Urinary catheter was removed on post-operative day 1 to aid early detection of neurological deficit. She remained asymptomatic and was discharged home on postoperative day 7 after her cardiovascular condition was optimized.

Consent was obtained from the patient and formally documented in her medical records allowing the reporting of relevant case details, investigations, and photos.

## 3 Discussion

This case illustrates the anesthetic challenges in balancing the risks and benefits of a particular anesthetic approach in a patient with PVOD.

PVOD is a rare condition with specific anesthetic considerations. It is classified as a subgroup of PAH and accounts for 5% to 10% of cases initially considered to be idiopathic PAH.^[1,2]^ Even though the first well-documented case of PVOD was described more than 70 years ago, the characteristics and pathophysiology of PVOD remain poor. PVOD preferentially affects the post-capillary pulmonary venules in contrast to idiopathic PAH which affects the small pre-capillary pulmonary arteries.^[2]^

It is important to differentiate PVOD from PAH due to its specific consideration for usage of pulmonary vasodilators. Patients with PVOD often have digital clubbing, more severe hypoxemia, significantly lower DLCO as compared to PAH. High-resolution computed tomography demonstrating features of interlobular septal thickening, mediastinal lymphadenopathy, and centrilobular ground glass opacities are highly suggestive of PVOD.^[1,3–6]^ Despite the histological differences, PVOD has a very similar clinical presentation to PAH but is characterized by a worse prognosis and the possibility of severe pulmonary edema with pulmonary vasodilators.^[2]^ The mean survival time for patients with PVOD is 2 to 3 years after diagnosis.^[4–6]^

The perioperative and anesthesia-related morbidity for patients with PAH undergoing non-cardiac surgery is quoted to be 27%.^[7]^ There is paucity of data on the surgical mortality and anesthetic management of patients with PVOD. General anesthesia (GA) may increase pulmonary vascular resistance leading to a pulmonary hypertensive crisis, right ventricular failure, and cardiopulmonary collapse. Her primary cardiologists in view of her admission for heart failure, quoted a 30% mortality risk if the procedure was done under GA.

While the use of pulmonary vasodilators is the gold standard in patients with PAH, the use of vasodilators for PVOD is controversial. A systemic review on use of vasodilators for treatment of PVOD showed improvement in 6-minute walk distance and pulmonary vascular resistance. However, pulmonary edema developed in most of these patients, with some being fatal.^[6]^ The dose of vasodilator if titrated slowly may avoid risk of pulmonary edema while achieving clinical effects.^[6]^ Slow titration of therapy may not be applicable in the perioperative setting for acute reversal of pulmonary hypertensive crisis. The risk of fatal pulmonary edema with usage of vasodilator therapy in an acute setting remains unknown. Hence, the anesthesia goal for management of patients with PVOD would be prevention of acute pulmonary hypertensive crisis as there is limited medical therapy available for acute reversal of the crisis, leading to cardiopulmonary collapse. Initiation of extracorporeal membrane oxygenation remains the last resort and has been used perioperatively for management of pulmonary hypertensive crisis and as interim measure while awaiting for definitive treatment with lung transplantation.^[8–10]^

Central neuraxial anesthesia if carefully conducted with calibrated regional anesthetic doses through combined spinal epidural technique, maintains systemic vascular resistance and avoids increase in pulmonary vascular resistance. It also provides excellent analgesia, thus avoiding the sympathetic response to surgical stimuli normally seen during GA. A CSE technique would facilitate a titratable anesthesia for the duration of her surgery. However, our patient's concurrent aspirin and anagrelide therapy posed her an increased risk of central neuraxial bleeding. Central neuraxial procedures on patients with concurrent aspirin and anagrelide have not been reported. There is evidence of major hemorrhagic events with concurrent use of aspirin and anagrelide.^[11–13]^ Our patient had significant bleeding from her CSE insertion site and she was monitored closely postoperatively. Post procedure management focuses on measures to reduce risk of central neuraxial bleeding and to facilitate early detection of central neuraxial hematoma in view of her significant epidural site bleeding.

In conclusion, PVOD has specific anesthetic considerations and it is crucial to differentiate PVOD from PAH. Due to its controversial use of vasodilators, there is limited medical treatment available for reversal of acute pulmonary hypertensive crisis. Hence, it is utmost important to prevent acute pulmonary hypertensive crisis in patients with PVOD.

## Author contributions

**Conceptualization:** Theng Wai Foong, Ashokka Balakrishnan.

**Writing – original draft:** Theng Wai Foong, Pei Shan Tan.

**Writing – review and editing:** Theng Wai Foong, Swapna Thampi, Ashokka Balakrishnan.
